# The Voluntary Hospitals Commission

**Published:** 1922-09

**Authors:** 


### THE VOLUNTARY HOSPITALS COMMISSION.

At the Conference between the Voluntary Hospitals Commission and representatives of the local Voluntary Hospital Committees, held at the Ministry of Health, Lord Onslow, who was in the chair, explained that the object of the Government, of the Commission, and of the local Committees was to maintain intact the voluntary system, and he had been charged by Sir Alfred Mond especially to emphasise this fundamental consideration. Their first duty was to administer the grant of £500,000 voted by the Government. To assist them in this work, the local Voluntary Hospital Committees had been established, and had rendered valuable assistance. In London the duties of a local Committee had been undertaken by King Edward's Hospital Fund, and the first emergency grants had been made by the Commission to certain London hospitals. In no case had the grant exceeded more than one-half of the estimated deficit on maintenance account for the year 1921. The estimate of Lord Cave's Committee of the deficit of the London hospitals was £463,000. King Edward's Fund had reported, however, that the actual deficit was £320,000. The Commission had made grants up to one-half of this—namely, £160,000 for London. In London a Combined Appeal for new money had been launched with marked success. In the rest of the country, 54 Voluntary Hospital Committees were operating, and there were only eight areas in which Committees had not yet been established. The return of deficits up to the present showed a lower figure than the estimate in Lord Cave's report, but the Chairman urged that all Committees should send in their claims without delay, in order that the Commission might be in possession of a final figure. It was anticipated that after paying one-half of the total deficits on maintenance accounts for 1921, the Commission would have a balance of about £125,000 in hand, and it was considering the allocation of such surplus. As regards the bulk allocation of the total sum at the Commission's disposal as between London and the provinces, it had resolved, subject to a final adjustment of the figures, that £225,000, including the amounts already voted, be appropriated to London, and £275,000, including the sums already voted, to the remaining areas. The information before the Commission went to show that the cost of maintenance was declining, while income was being maintained and in many cases rising.

The Conference passed unanimously the following resolution :—" That there should be established a Standing Committee of representatives of the local Hospital Committees, to whom the Commission could refer for advice and assistance, and that the personnel in such Committee should include representatives of the less populous districts. The Committee to be appointed in the first instance by the Commission."

Mr. H. N. Crouch (Somerset) spoke of the difficulties in securing co-ordination and economies, because the hospitals were apt to regard themselves as isolated units. The lack of system tended to waste in accommodation and in the medical services. In Somerset the total cost of treating residents at the various hospitals was represented by 2s. 11d. per head of population, of which 1s. 1d. is spent in Bristol and Bath, and 1s. 10d. in the rest of the country. The burden of finding the money, however, was unevenly distributed. A district that provided no hospital found about 7d. per head, whereas a district with a fully-equipped hospital bore the cost at the rate of 3s. 1d. per head. It was essential to have the Hospital Committee areas based on areas common to the large city hospital and the county area which it served ; his suggestion was based on the scheme outlined in Lord Dawson's report. He proposed the following resolution :—" That the Voluntary Hospitals areas be so regrouped that each area constitute a suitable unit in which to make the attempt to effect co-ordination between existing hospitals and to gradually create a complete and properly organised hospital system on a voluntary system."

Lord Onslow suggested that this resolution should be referred to the local Voluntary Hospital Committees for their views, and that when these were obtained the Commission could consider the matter with the assistance of the Consultative Committee.

The representative of the North Wales Committee referred to the difficulties of his Committee in regard to administration expenses, seeing that they covered an area of six counties.

Lord Cave explained that his Committee did not recommend that assistance from public funds should be given to the local Voluntary Hospitals Committee as a right, because this would have introduced public management into the scheme. It had suggested that local authorities should have the power to contribute, if they so desired. The Surrey County Council had provided a sum for the Surrey Local Voluntary Hospital Committee, and he hoped that the latter would respond by furnishing the County Council each year with a report on the work done. Apart from contributions from a local authority, there appeared to remain only subscriptions from voluntary sources.

In the subsequent discussion, it appeared that the majority of local authorities had contributed to the expenses of the local Committees. The representatives of Cornwall and Dorset spoke against any assistance from public funds. The Chairman of the Derbyshire Voluntary Hospital Committee, who is also Chairman of the County Council, thought that there would be no difficulties with a County Council if properly approached. Lord Onslow explained that this was a matter which each Committee must settle for itself. There was no compulsion on the local Voluntary Hospital Committee to ask, or on the County Council to give, financial assistance for the administration expenses of these Committees ; but if the local authority desired to respond to such a request, the payment would receive the sanction of the Ministry of Health, which was required in such a case.

With regard to the difficulties which local Committees experience in obtaining information on the methods pursued in other areas, Mr. Davis (Manchester) suggested that the Commission should issue a pamphlet on the methods which have secured the high standard of excellence in some of the best hospitals, giving particulars of the control and administration of the hospital, how it reaches the public, how it obtains contributions, and how the public regard it. Lord Onslow said it was apparently desired that the Commission should issue a questionnaire on these points, tabulate the answers and issue a summary. It was unanimously agreed that this should be done.

Lord Onslow moved the following resolution, which was intended for the guidance of local authorities: "That local hospital committees should not recommend any extension scheme to the public, unless they are satisfied (*a*) that the extension is necessary and is planned on a reasonable scale, and that the services cannot be provided by co-operation with other institutions; (*b*) that sufficient funds will be available within a reasonable time for the execution of the scheme or an effective part of it; and (*c*) that funds are or will be available to meet the cost of the maintenance of the hospital, including any additional maintenance charges resulting from the extension, and that the appeal for capital expenditure will not prejudice the raising of the funds required for the maintenance of the hospital itself or other institutions in the area." He explained that no part of the Government grant was available for capital expenditure for extensions. After the grants based on deficits for 1920 had been paid, the balance would be distributed to meet accumulated deficits. The resolution embodied three conditions which should be fulfilled before the L.V.H.C. recommended any extension, the object being to secure that funds for extension are not raised in circumstances detrimental to the raising of the essential funds for maintenance purposes. The resolution was carried unanimously.

With regard to the administration of the Government grant, Mr. Miller (Devon) urged that a period of three years should be adopted as the basis for calculating the deficiency of any hospital, as it would gauge fairly the real financial position. Mr. Maynard (King Edward's Fund) pointed out that the grant administered by the Commission was an ambulance grant. He suggested that the test of a hospital's position was not the deficit in any individual year, but the relation between liabilities resulting from deficits, and the available assets. The Commission should have special regard to hospitals which had exceeded their borrowing powers.

Lord Cave observed that the object of the fund was not to reward merit, but to assist disabled institutions. He thought, however, that, subject to the prior claim of the deficits of 1921, any surplus could be fairly devoted to accumulated deficits, and he agreed that preference should be given to institutions showing a real deficit—*i.e.*, a loss which had resulted in a deficiency of assets as against liabilities.

Mr. Davis (Manchester), speaking of the need for systematic collection from employers and workers, stated that the public was anxious to maintain voluntary hospitals. The difficulty was to create facilities through which help could be given. In particular the crux lay in getting to the employee through the employer, who did not always facilitate the entrée to his works. He suggested that the Commission should approach the Federation of British Industries, and urged that steps should be taken to obtain the sympathy of Labour leaders, and to secure an expression of opinion from them that they are not averse from the payment of weekly contributions by workpeople. Alderman Sheppard (Bristol) thought that local committees could exercise more influence with employers in their own localities than could the Commission. While the personal opinion of Labour leaders was probably in favour of the voluntary system, their political view was in favour of nationalisation of hospitals. It was desirable to keep clear of the political atmosphere, and to be in direct touch with employers and employed.

Mr. Osborn reported on the work done by the Sheffield Joint Hospital Council. The basis of their scheme was to secure 1d. in the pound of wages paid, plus one-third added by the employer. In 1922 they had raised up to date £28,000. The scheme covered over 1,000 factories and 120,000 contributors. Mr. Cozens-Hardy (Liverpool) gave particulars of the comparison which he had made of several schemes, showing generally that the contributions amounted to 5s. per head of the insured population, or 2s. per head of the total population. The most important thing was to deduct the contribution before the wages were paid. He agreed that it was inadvisable to approach the Labour Party officially, and questioned whether it was desirable to ask for a fixed contribution from employers, who are always asked to contribute to special funds for extensions and so forth. Mr. Barnes (Derbyshire) referred to the successful scheme of contributions from employers and employed in force for 20 years in Chesterfield Royal Hospital. In 1921 the income balanced the expenditure. Twelve representatives of the workpeople were members of the board, which consisted of 17, plus ex officio members. Mr. Drury (Norfolk) said that in his county they had secured the assistance of the trade unions in their contributory scheme.

Sir Walter Kinnear gave an outline of what had happened in connection with the valuation of the Approved Societies. Before the results were declared the financial difficulties of the hospitals had become apparent to the Department. There was no provision in the Acts for any money from the funds of the societies to be allocated to the hospitals, and in 1920 the Ministry took legislative sanction in order to enable the societies to release some of their surplus funds. They then circularised every society, advocating the claims of the hospitals, and suggesting that the Approved Societies should seriously consider the desirability of allocating a portion of the surplus for payment of members when in hospital, pointing out that the societies benefited from the services which they received from the hospitals in shortening the duration of the illnesses of their members. The Approved Societies considered the matter, and the Department had a number of conferences with them. Unfortunately the issue of the valuation reports synchronised with a period when the cost of living was high, and, having regard to the fact that the Act provides sick pay only at the rate of 15s. a week for men and 12s. for women, the majority of the Approved Societies decided to devote the total amount of their surpluses to additions of 2s. to 3s. a week to the cash benefits. That decision was entirely within their own right, and the Department had no power to interfere with it. The Department succeeded in getting 976 societies and branches to devote a portion of their surpluses to hospital provision, and schemes to that effect had been approved for five years—that is, up to the issue of the next valuation results. The schemes had freed something like £200,000 a year for payments for particular members of those Approved Societies in hospitals.

The scheme of payment was hampered by certain encumbrances. The first difficulty was that payment must be made to the hospital where the member received treatment and in respect of a particular member, because only members who were in the Approved Society during the time when the surplus was earned were entitled to participate in this additional hospital provision. There had been conferences with the British Hospitals Association, and a scheme had been set up asking that the hospitals who received patients from such Approved Societies as pay during residence should communicate to their societies the name of the patient, his address, his number in the society, the date of arrival in hospital and date of discharge from hospital. For a variety of reasons the scheme was not working very well. Only about £30,000 had been paid out, although a considerable sum of money had accrued. The societies were anxious to facilitate the disbursement of the money to the hospitals, but they wanted accurate information. The speaker had recently surveyed the accounts of a society with a membership of over 2,000,000 in England and Wales; for the first quarter of 1922 it received returns from only 92 hospitals, and these were incomplete. In many cases simply the name of the patient was given, and it was difficult to trace him and see if he was a member for whom a payment could be made. Another difficulty was that the Approved Society gave the member a voucher to give to the hospital, so that the hospital might identify the member as a person for whom it would be paid. Insured persons were reluctant in many cases to give up the vouchers, thinking that the fact that the society was paying for them would in some way militate against their sick pay, which, of course, was quite erroneous. Also some of the hospitals were inclined to think that the receipt of these comparatively small sums from the Approved Societies would discourage voluntary contributions. The Department would welcome any suggestions which would help to get the machine into better order.

Mr. Wiltshire (Birmingham) spoke of the difficulties of securing adequate contributions from areas which had no hospital facilities of their own, and Dr. Flemming (Wilts) urged that collections should be for areas, and not for individual hospitals, provided that the areas were so divided that the incentive of local patriotism was not lost. Mr. Cook (Bristol) said that in 1921 the Bristol Royal Infirmary treated 5,368 patients, of whom only 2,372 came from Bristol itself. The contributions per head amounted to £5 10s. from Bristol, and £4 10s. from the outside area.

Mr. Brock, the Secretary of the Commission, said that information was being collected as to the possibilities of co-operative purchase, and would be communicated to the Local Committees if found to be valuable. Information on this subject from the Local Committees would be welcomed.

Lord Cave inquired whether the Local Committees would be expected to continue their work, and for how long. He hoped that they would continue, since after the distribution of the grant their first duty would be to co-ordinate the hospitals in their areas. They could help materially in the collection of funds. He agreed that workpeople should be approached locally and individually in this matter, and referred to the importance of keeping hospital accounts on a simplified uniform system. He thought there would be advantages in the Commission's continuance in some form, but that nothing should be done which would militate against the complete preservation of the voluntary system.

Lord Onslow considered that the Local Committees would have so much to do that, at least for some time to come, they could not dissolve, and hoped that their usefulness would secure for them a permanent place in the hospital system. As to the Commission, some central body must continue to act as a liaison between all the Local Committees. It was important that voluntary hospitals should keep in touch with the Government, in view of the various public services for which they were receiving grants. It was partly for this reason that the local public health authorities had representation on the Local Committees. With necessary modifications the Commission might become a permanent part of our hospital system, but its sole function would be to maintain intact the voluntary principle, and if it were calculated to lead either to municipalisation or nationalisation, he would like to see its immediate demise.

[The Commission has decided that unless exceptional circumstances can be shown, it will be unable to consider any claims in respect of 1921 deficits which are received later than November 30 next. Local Committees should notify all hospitals within their area that any application should be sent in at once.—Ed.]

---

### THE SECRETARYSHIP OF THE POPLAR HOSPITAL.

Mr. Rogers, who retired from the post of secretary of the Poplar Hospital a short time ago, has just died. It seems to us unfair that a new secretary has been appointed who has no hospital experience, whereas the assistant secretary, a man of middle age, who has been connected with the hospital for a number of years, has been passed over. From the general standpoint also the course followed is to be deprecated. If the best is to be got out of the hospital service, its members ought to be able to look forward to promotion in due course.

